# New Arrangements

**Published:** 1859-03

**Authors:** 


*New Arrangements.*—The publisher has lately been able to make arrangements with the enterprising house of Baillière, and with B. Westermann & Co., of New York, by which we will be enabled to receive with regularity, French, German, British, and Spanish Journals, as well as to order, with facility, medical works published in those countries. This will be a material improvement in our Journal, which we will spare no trouble to make as good in foreign news as any of its class in the country, as we will publish occasional translations from these periodicals.

